# Evaluation of Remote Photoplethysmography Measurement Conditions toward Telemedicine Applications

**DOI:** 10.3390/s21248357

**Published:** 2021-12-14

**Authors:** Akito Tohma, Maho Nishikawa, Takuya Hashimoto, Yoichi Yamazaki, Guanghao Sun

**Affiliations:** 1 Department of Mechanical Engineering, Tokyo University of Science, Tokyo 162-8601, Japan; 4520553@ed.tus.ac.jp; 2 Graduate School of Informatics and Engineering, The University of Electro-Communications, Tokyo 182-0033, Japan; nishikawa@secure.ee.uec.ac.jp (M.N.); guanghao.sun@uec.ac.jp (G.S.); 3 Department of Home Electronics, Kanagawa Institute of Technology, Kanagawa 243-0292, Japan; yamazaki@he.kanagawa-it.ac.jp

**Keywords:** telemedicine, remote photoplethysmography (rPPG), blood volume pulse, heart rate variability (HRV), non-contact, remote sensing

## Abstract

Camera-based remote photoplethysmography (rPPG) is a low-cost and casual non-contact heart rate measurement method suitable for telemedicine. Several factors affect the accuracy of measuring the heart rate and heart rate variability (HRV) using rPPG despite HRV being an important indicator for healthcare monitoring. This study aimed to investigate the appropriate setup for precise HRV measurements using rPPG while considering the effects of possible factors including illumination, direction of the light, frame rate of the camera, and body motion. In the lighting conditions experiment, the smallest mean absolute R–R interval (RRI) error was obtained when light greater than 500 lux was cast from the front (among the following conditions—illuminance: 100, 300, 500, and 700 lux; directions: front, top, and front and top). In addition, the RRI and HRV were measured with sufficient accuracy at frame rates above 30 fps. The accuracy of the HRV measurement was greatly reduced when the body motion was not constrained; thus, it is necessary to limit the body motion, especially the head motion, in an actual telemedicine situation. The results of this study can act as guidelines for setting up the shooting environment and camera settings for rPPG use in telemedicine.

## 1. Introduction

Various telemedicine services have garnered interest with the development of information and communication technology (ICT) [1,2]. In addition, the COVID-19 pandemic has increased the demand for telemedicine due to the risk of infection in physical hospitals. Several types of telemedicine services using biological signals, such as blood pressure [3], blood glucose [4], ECG, SpO2, and temperature [5] have been proposed as healthcare indicators to prevent critical illness and frailty. For example, Yamazaki et al. [3] proposed a robotic system to monitor the blood pressure and heart rate of a patient at home using a sphygmomanometer. In addition, Glooko, Inc. [4] proposed a cloud service that manages blood glucose meters and insulin doses for diabetic patients and is used for in-hospital consultations and remote guidance. Alternatively, Abo-Zahhad et al. [5] proposed a wireless emergency telemedicine system that continuously collects and evaluates biological signals; if abnormal values are encountered, physiological data are immediately submitted to a remote medical server via both the cellular network and the Internet. In particular, the heart rate (HR) and heart rate variability (HRV) are important indicators of physical and mental illness because they are modulated by the autonomic nervous system, autonomic nerve response [6], and mental stress [7]. To monitor the HRV, remote photoplethysmogram techniques [8,9,10], rPPG for short, are expected to be a casual non-contact method that can be implemented in telemedicine services because they do not require dedicated wearable devices or electrode pads required for photoplethysmogram (PPG) [11] and electrocardiogram (ECG) measurements. In fact, the heart rate and its variability obtained by rPPG are used as indicators for patient monitoring [12], sleep monitoring [13], and neonate monitoring [14].

Despite having advantages for telemedicine services, the error in the HRV obtained through rPPG increases due to several factors, such as lighting conditions [15], body motion [16], and camera parameters [17]. Accordingly, one of the major concerns is to establish a method to suppress the influence of these factors for achieving highly accurate rPPG measurements [18,19,20]. To improve the quality of the rPPG, a blind source separation (BSS) algorithm [18] was introduced. For example, independent component analysis (ICA), which seeks the source signals that are maximally independent in a statistical sense, is commonly used as a BSS algorithm and is feasible for HR measurement but requires a long-time series signal and cannot be expected to achieve high performance. In contrast to BSS, a model-based method that uses the pre-knowledge of different color components is also proposed. The chrominance-based method, named CHROM, proposed by De Hann et al. [19], considers diffuse reflection, which relates to pulsation, and specular reflection, which does not pertain to pulse signal, which together make the color variations. Therefore, they use a linear combination of chrominance features to remove specular reflection by assuming a standardized skin color to white-balance the images. Furthermore, the blood volume pulse signature (PBV) method proposed by De Hann et al. [20] uses the unique signature defined by the absorption spectrum of hemoglobin and retrieves the pulse by restricting all color variations to the pulsatile direction. While these efforts are underway, they have not yet been able to scale up from the laboratory to an actual situation, such as telemedicine services, because it is difficult to completely eliminate the effects of body motion and lighting.

The purpose of this study was to build up knowledge about the constraints and limitations of measuring HR and HRV accurately by rPPG, which would provide guidelines for preparing the camera settings and shooting environment. Therefore, we investigated an effective, appropriate setup considering illumination, camera parameters, and body motion to achieve precise HRV measurements by rPPG in telemedicine situations. First, in terms of illumination, the light source should emit sufficient energy, because the amplitude of the blood pulse is proportional to the intensity of illumination [21]; that is, the higher the light intensity, the stronger the measured pulse signal. However, if the illumination is too high, image clipping may occur. In addition, the direction of illumination [21] is important because the dark and bright areas of the face change due to its uneven surface. Second, the frame rate of the camera should be carefully considered. A lower frame rate causes a larger error in detecting the blood pulse interval (R–R interval or RRI) [22]. As a result, the accuracy of the HRV measurement may be affected by the frame rate because it is derived from the frequency analysis of the RRI. Third, considering the illumination condition, it is necessary to suppress body motion because it changes the direction of the camera and light source, resulting in the fluctuation of shade on the face. Therefore, the development of effective pulse extraction methods that compensate body movements is one of the main goals in rPPG studies [23,24]. One robust rPPG method [23] divides the whole face into many small regions and combines the different regions with weights to reduce the effect of body movements and measure the HR. However, the extent to which body motion is involved in accurate HRV measurements has not been investigated. In the experiment, various conditions concerning illumination, the direction of light, the frame rate of the camera, and body motion were examined to verify the accuracy of the RRI and HRV measurements.

## 2. Materials and Methods

### 2.1. Measurement Equipment

A single RGB camera (DFK33UX287, The Imaging Source Asia Co., Ltd., Taipei City, Taiwan) with a maximum frame rate of 539 fps was used for the rPPG measurements. The camera was connected to a PC via USB 3.0, and the video image was captured at 720 × 540 resolution and saved in an uncompressed format. In addition, a contact-type ECG sensor (LRR-03, GMS Co., Ltd., Yangju-si, Korea) was used to measure the blood pulse simultaneously with the measurement using the camera for comparison. The signal of the ECG sensor was recorded at a sampling rate of 100 Hz using a 16-bit A/D converter. Only the onset and offset times of the rPPG and ECG were synchronized because the sampling rates of these sensors differ.

### 2.2. Experimental Setup and Protocol

**Figure 1:**
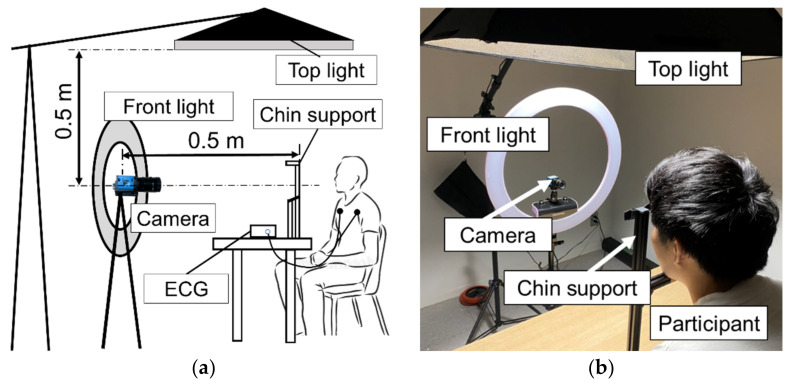
Experimental setup: (**a**) overview and (**b**) scene of the experiment.

Figure 1 shows the experimental setup. The camera was placed at a distance of 0.5 m from the subject, as done by Verkryusee et al. [8]. The subject was instructed to sit and look at the camera. Additionally, to suppress head (body) motion, the subject was asked to put his chin on the head fixation apparatus. The height and orientation of the camera were adjusted so that the entire face was within the angle of view. The subject’s face was illuminated from the front and/or top by LED lights of the same wavelength (white color). Incidentally, to prevent external light from entering, the experiment was conducted in a room with no windows. In the experiment, three healthy males (21–22 years old) participated as volunteers. Written consent was obtained from the participants before the experiment, and the experimental procedures were approved by the ethics committee of the institution.

To obtain the optimal rPPG conditions, the accuracy of RRI or HRV was evaluated through experiments under three different conditions. A detailed description of each condition is provided below.

#### 2.2.1. Experiment on Effect of Light Source

The first experimental condition was set up to investigate the effect of illumination on HRV measurements. Specifically, the experiment was conducted by changing the direction and intensity of the light source. With respect to the light source direction, it had three conditions: front, top, and front and top. In addition, the illuminance was adjusted to 4 degrees: 100, 300, 500, and 700 lux per light source direction. Considering all combinations of the three lighting directions (front, top, and front and top) and four illuminance conditions (100, 300, 500, and 700 lux), we investigated 12 different light source conditions in total. Here, the condition of 500 lux from the top is the standard for a typical room, and the captured image is overexposed when it exceeds 700 lux. Incidentally, the frame rate of the camera was set to 30 fps, and the measurement time was 2 min. In this experiment, the measurement accuracy of the RRI, which is used to derive HRV, was evaluated.

#### 2.2.2. Experiment on the Effect of Camera’s Frame Rate

After determining the preferred light source condition, the effect of the frame rate of the camera on the HRV measurement was examined. For this purpose, the experiment was conducted with four different frame rates under fixed light source conditions: 15, 30, 60, and 100 fps. Here, 30 fps is the standard frame rate of a typical camera. Additionally, the aperture value of the camera was adjusted so that the brightness of the image would be constant regardless of the frame rate because the amount of light received by the image sensors decreases as the frame rate increases. In this experiment, the accuracy of three types of HRV metrics (LF, HF, and LF/HF) was evaluated. To obtain reliable HRV values, the measurement time was set as 6 min because the HR measurement guideline [22] mandates a measurement time of at least 2–5 min to calculate the HRV.

#### 2.2.3. Experiment on the Effect of Body Motion

The effect of body motion on the HRV measurement should be evaluated because it is difficult to constrict the user’s behavior when assuming the actual situation of telemedicine services. Here, it is thought that body motion includes not only head motion but also minute motions, such as facial and lip movements, and breathing. As mentioned before, the direction (angle) of the light source to the skin and to the camera are changed when the face moves significantly, which affects the extraction of the blood pulse. Additionally, small facial movements, such as movements of muscles around the mouth during speaking, also affect the light reflection on the facial skin. As a result, it appears as a noise component of blood pulse. To evaluate the effect of such body motions, three types of conditions were considered: fixation, non-fixation, and non-fixation and speaking. In the fixation condition, the subject’s head was constricted using the fixation apparatus, as in the previous experiment. Meanwhile, in the non-fixation condition, the subject was instructed to look at the camera without head constriction. In the non-fixation and speaking condition, the subject was also asked to respond to questions from the experimenter based on the medical interview sheet in addition to the non-fixation condition. The other experimental conditions including the illumination, the lighting direction, and the frame rate were defined as the appropriate conditions and values derived in the experiment described in Section 2.2.1 and Section 2.2.2. Additionally, the accuracy of the HRV metrics (LF, HF, LF/HF) was evaluated for each condition.

### 2.3. Image and Signal Processing

**Figure 2:**
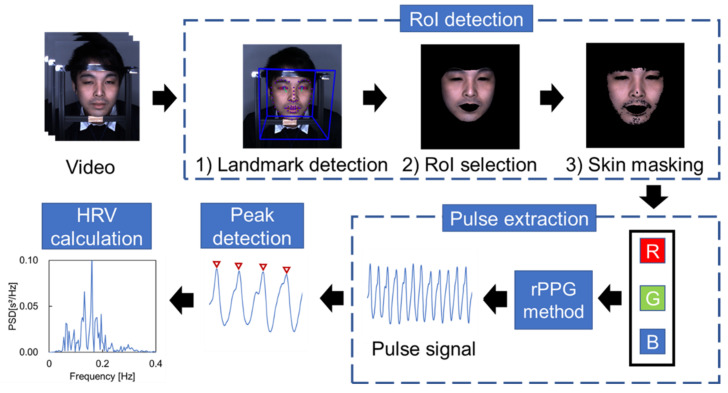
Overview of the processing flow.

Figure 2 shows an overview of the entire processing flow used to obtain the HRV measurements. The process comprises four steps: region of interest (RoI) detection, pulse extraction, peak detection, and HRV calculation. A detailed description of each step is provided below.

#### 2.3.1. Region of Interest (RoI) Detection

OpenFace [25], an open-source facial video analysis toolset, was used to obtain two-dimensional landmarks of the face in pixels for each frame of the captured video. In the RoI selection, the areas of the eyes and the mouth are eliminated using the landmarks. Subsequently, each image frame is converted to the HSV color space [26] to extract the skin color region and removal the hair and beard regions.

#### 2.3.2. Pulse Extraction

This process extracts the pulse signal from color variations in the skin color regions of the RoI. Prior to applying the pulse extraction algorithm (rPPG algorithm), a spatial average of the pixel values in the skin color regions was calculated in every frame and decomposed into RGB components. Subsequently, to compensate for the variability of the sampling rate, the resampling process was performed for each signal using linear interpolation. Here, the RGB components obtained are expressed as $x={\left[\begin{array}{ccc} x_{r}\left(t\right) & x_{g}\left(t\right) & x_{b}\left(t\right) \end{array}\right]}^{T}$. Then, the rPPG algorithm was applied to extract the pulse signal from x. In this study, we adopted two rPPG algorithms for comparison: the Green and POS methods. The details of each algorithm are presented below.

The Green method is a single-wavelength method [27,28] which is a well-known algorithm and is still popular owing to its simplicity and low computational cost. This method obtains the pulse wave signal by detecting the fluctuation of the green component of the skin color region, which is extracted from the RoI, as hemoglobin absorbs green light. In this study, the pulse wave is obtained by calculating the average of the green component $x_{g}\left(t\right)$ of the RoI in the pulse extraction process, as shown in Figure 2. Additionally, a 0.7–2.5 Hz bandpass filter is applied to the signal to remove noise.

The POS [24] method uses a plane orthogonal to the skin tone (POS) for pulse signal extraction, and it can perform blood pulse extraction while suppressing the effects of body motion and light source. First, a time window of 1.6 s was applied with a step size of one frame to remove the trend of RGB components $x={\left[\begin{array}{ccc} x_{r}\left(t\right) & x_{g}\left(t\right) & x_{b}\left(t\right) \end{array}\right]}^{T}$, where the standardized signals, $\bar{x}={\left[\begin{array}{ccc} {\bar{x}}_{r}\left(t\right) & {\bar{x}}_{g}\left(t\right) & {\bar{x}}_{b}\left(t\right) \end{array}\right]}^{T}$, are obtained by dividing x by the average value in each time window. Additionally, a 0.7–2.5 Hz bandpass filter is applied to remove the noise component, as in the Green method. Then, the two-dimensional signal $S={\left[\begin{array}{cc} S_{1}\left(t\right) & S_{2}\left(t\right) \end{array}\right]}^{T}$ is calculated from the standardized signal $\bar{x}$ using Equations (1) and (2).

(1) $$S_{1}\left(t\right)={\bar{x}}_{g}\left(t\right)-{\bar{x}}_{b}\left(t\right)$$

(2) $$S_{2}\left(t\right)=-2{\bar{x}}_{r}\left(t\right)+{\bar{x}}_{g}\left(t\right)+{\bar{x}}_{b}\left(t\right)$$

Subsequently, $h\left(t\right)$ is calculated from $S_{1}\left(t\right)$ and $S_{2}\left(t\right)$ using Equation (3).

(3) $$h\left(t\right)=S_{1}\left(t\right)+\frac{\sigma \left(S_{1}\right)}{\sigma \left(S_{2}\right)}\cdot S_{2}\left(t\right)$$

Finally, the pulse signal can be obtained by summing $h\left(t\right)$ with a shift of one frame.

#### 2.3.3. Peak Detection

**Figure 3:**
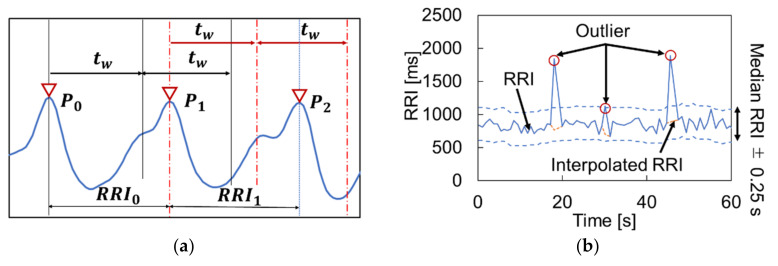
Processing procedure: (**a**) peak detection procedure and (**b**) peak correction.

Prior to peak detection, the pulse signal is interpolated to 250 Hz using spline interpolation, because a low sampling rate potentially causes jitter in the peaks and changes the spectrum [22]. Then, as shown in Figure 3a, peak detection is performed by overlapping the time windows tw, which are defined by the following Equation (4).

(4) $$tw=\left(\frac{1}{f_{c}}\right)\times 0.7$$

Here, tw is the length of the time window, and $f_{c}$ is the frequency that appears to be the main frequency component of the blood pulse. The frequency $f_{c}$, which takes the strongest power in the frequency band between 0.80 and 1.65 Hz, is defined by FFT analysis. The first step of the peak detection process is to detect the first maximum value between 0 and tw as $P_{0}$. Subsequently, it detects the peak in the next time window as $P_{1}$. By continuing this process until the end with a time shift, the time series RRI is obtained.

#### 2.3.4. HRV Calculation

First, the outliers are removed from the time series RRI, as they affect the HRV calculation. Here, the median value of every 10 plots of RRI is calculated with the shift of one plot, and the median RRI ± 0.25 s is used as the threshold [29]. The outliers exceeding the threshold were removed and modified by spline interpolation, as shown in Figure 3b. In addition, the time series RRI was resampled again at 4 Hz using spline interpolation, referring to [22], because the data intervals of RRI were unequal. Then, to analyze the fluctuation of RRI, known as HRV, we computed the short-time FFT of the 2-min (480 samples) Hamming sliding window with the 15-s (60 samples) increment. Here, the length of the sliding window was defined by referring to the guideline [22] to obtain reliable HRV values. Finally, as a result of this spectral analysis, three critical frequency domain parameters were obtained: very low frequency (VLF, 0.0033–0.04 Hz) power, low frequency (LF, 0.04–0.15 Hz) power, and high frequency (HF, 0.15–0.4 Hz) power. These were quantified in normalized units to minimize the impact of differences in total power. LF and HF are known to reflect the parasympathetic and sympathetic activities of the autonomic nervous system [20]. Furthermore, the ratio of these power spectra, LF/HF, is widely used as an indicator to quantitatively evaluate autonomic activity.

## 3. Results

### 3.1. Effect of Light Source

To investigate the effect of light source, we evaluated the mean absolute error (MAE) of the RRI. For each peak i, we compared the estimated RRI ($RRI_{i})$ with the reference RRI ($RRI_{i}^{ECG}$). The following Equation (5) was used to calculate the MAE. Here, N is the number of peaks.

(5) $$\mathrm{MAE}=\frac{1}{N-1}\overset{N-1}{\underset{i=1}{\sum }}\left|RRI_{i}-RRI_{i}^{ECG}\right|$$

**Figure 4:**
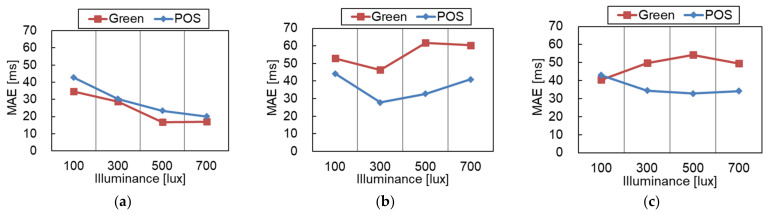
Mean absolute error (MAE) of RRI under different illuminance directions: (**a**) FR (Front), (**b**) TO (Top), and (**c**) FR-TO (Front and Top).

Figure 4 shows the MAE of the RRI obtained under different light source conditions for both rPPG methods (Green and POS). In the following, the three different light source directions are denoted as FR, TO, and FR-TO.

A comparison of the light source directions shows that the MAE of FR is smaller than those of TO and FR-TO in both rPPG methods. Additionally, the comparison of the rPPG method shows that the MAE of the POS method is smaller than that of the Green method under TO and FR-TO conditions, which indicates that the POS method is less affected by the light source direction.

In the FR condition, the MAE tends to be lower when the illuminance increases. This is because the effect of noise becomes relatively small as the amplitude of the pulse signal increases as the amount of light increases. On the other hand, the TO and FR-TO conditions did not show such a tendency for either rPPG method.

The experimental results indicate that the FR condition exhibits lower MAEs in every illuminance condition regardless of the rPPG method, and MAEs decrease to the minimum over 500 lux. Therefore, the appropriate light source condition is the FR condition of 500 lux.

### 3.2. Effects of Camera’s Frame Rate

**Figure 5:**
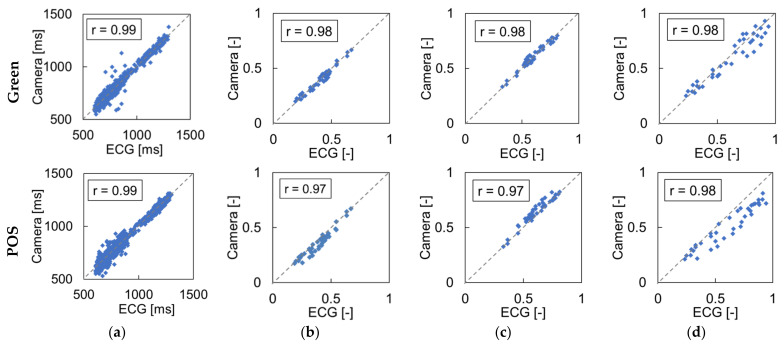
Comparison between rPPG and ECG in RRI and HRV metrics, LF, HF, and LF/HF at 30 fps. The horizontal axis is the result obtained by the ECG, and the vertical axis is the result obtained from the camera. The upper four graphs show the results of the Green method and the lower four graphs show those of POS method: (**a**) RRI, (**b**) LF, (**c**) HF, and (**d**) LF/HF.

The experiment for evaluating the effect of the frame rate of the camera was conducted under the appropriate light source condition, that is, the FR condition with 500-lux illuminance, and both rPPG methods (Green and POS) were used to extract the blood pulse. Figure 5 shows the correlations between both rPPG methods and ECG in RRI and three HRV metrics (LF, HF, and LF/HF) obtained at a frame rate of 30 fps as one of the experimental results. The results showed that the correlations were approximately 90% for all metrics in both rPPG methods.

**Figure 6:**
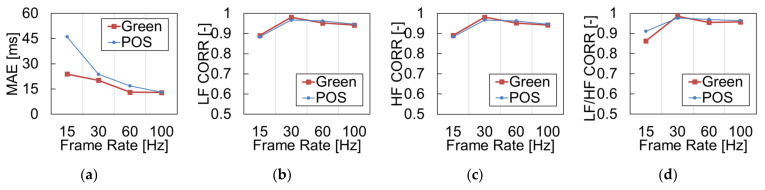
Performance metrics of each parameter: (**a**) RRI, (**b**) LF, (**c**) HF, and (**d**) LF/HF.

Figure 6 shows the mean absolute error of RRI and the correlation coefficients of HRV metrics (LF, HF, LF/HF) obtained under different frame rates. The error of the RRI decreased as the frame rate was increased for both rPPG methods. The correlation coefficients of the HRV metrics, LF, HF, and LF/HF, were lower at under 15 fps than those of higher frame rate conditions, but the coefficients changed slightly above 30 fps. The reason for this is that it is difficult to detect a slight fluctuation in the RRI when the temporal resolution decreases owing to the decrease in the frame rate.

### 3.3. Effects of Body Motion

**Figure 7:**
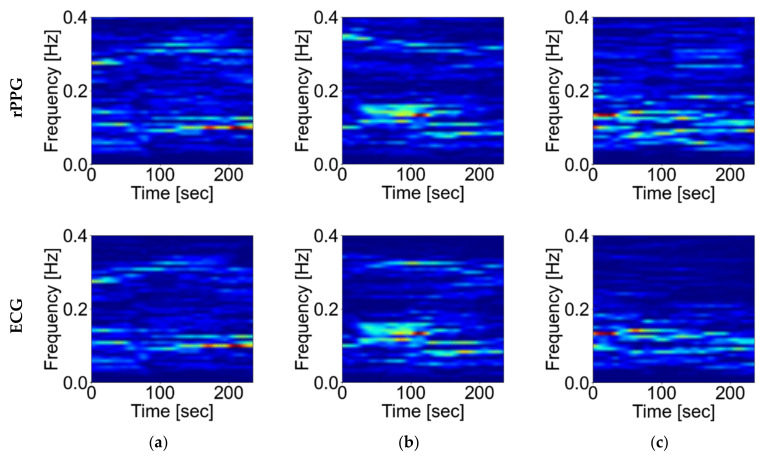
HRV Spectrum. The upper three graphs are estimated values. The lower three graphs are taken from measurements: (**a**) FIX (fixation), (**b**) FIX (non-fixation, stationary), and (**c**) SPK (speaking).

The experiment was conducted under the appropriate light source and frame rate conditions derived in the previous experiments, that is, the FR condition with an illuminance of 500 lux and a frame rate of 30 fps. The effect of body motion was evaluated based on the HRV spectrum to examine changes in HRV spectral analysis during body movement. In the spectral analysis, the short-time FFT of the time series RRI was computed using the 2-min (480 samples) Hamming sliding window with the 15-s (60 samples) increment. Figure 7 compares the spectrum acquired by the POS method, whose robustness against body movements has been verified in previous studies [22], and the ECG in the fixation, non-fixation, and non-fixation and speaking conditions. These different conditions are denoted as FIX, nFIX, and SPK. In the FIX and nFIX conditions, the spectrograms of the rPPG are close to those of the ECG. However, there is a small amount of noise in the SPK condition.

Table 1 summarizes the MAE of the RRI and the correlation coefficients of the HRV metrics. The results show that the MAE of the RRI in SPK was approximately twice as large as that of FIX and nFIX, and the correlation coefficients of the HRV metrics of SPK were much lower. In addition, the comparison between FIX and nFIX shows that the correlation coefficient of LF/HF is approximately 0.30 lower. As a result, the MAE was larger, and the correlation was lower in the nFIX and SPK conditions with body motion. A possible reason for this is that body motion reduces the accuracy of face tracking. In addition, it is thought that light reflection from the skin also changes with motion, and such color distortion appears as noise in the pulse signal and causes a decrease in accuracy.

## 4. Discussion

### 4.1. Influence of Light Source

Appropriate light source conditions were experimentally investigated for telemedicine services. The experimental results showed that the accuracy of the RRI measurement with rPPG was affected by the direction and illuminance of the light source. As for the direction of the light source, the accuracy of RRI extraction is better when illuminated from the front than from the top or from the top and front. This is because specular reflections on the skin surface due to the change of light source direction cause different rPPG strengths in RGB components. This result shows an agreement with the study by Wang et al. [30] which indicated that the signal-to-noise ratio (S/N) of a single light source was higher than that of multiple light sources due to the different color changes in the facial area. Therefore, in general environments with many light sources, such as offices and homes, it is desirable to either turn off the lights or block the external light in addition to preparing a light source in front of the face.

In terms of illuminance, for the FR condition, the accuracy is minimized under low illuminance (100 lux). The reason for this is that the amplitude of the pulse signal caused by the heartbeat becomes smaller and the camera noise becomes larger in a low-illuminance environment. In fact, Wang et al. [30] also noted that the signal-to-noise ratio (S/N) decreases at low light levels. To address this problem, near-infrared (NIR) remote photoplethysmography (PPG) [31] is thought to be an effective method because it uses infrared light.

### 4.2. Influence of Camera’s Frame Rate

Figure 6 indicates that the MAE of the RRI decreases as the frame rate increases. This is thought to be due to the time difference between frames. For example, because the time interval between adjacent frames is 33.3 ms in the case of 30 fps, the standard error of the R-peak can be theoretically ±16.6 ms. On the other hand, the standard deviation of the R-peak can be ±5 ms for 100 fps. In addition, the sampling rate was adjusted to 250 Hz using spline interpolation for the detection of pulse signal peaks; however, the complemented peaks did not perfectly match the actual peaks. In the measurement of HRV, the correlation coefficients of LF, HF, and LF/HF exceed 90% at 30 fps or higher, which indicates that the variation in HRV can be accurately estimated. Furthermore, in previous studies investigating the effect of frame rate [32], it was shown that even if the frame rate was reduced to 20 fps, the effect of the frame rate on HRV was not significant. Therefore, it is thought that the appropriate frame rate of the camera is 30 fps or higher when measuring HRV.

### 4.3. Influence of Body Motion

From the experimental results in Table 1, it is found that a large motion artifact appears in the nFIX and SPK. Therefore, in order to conduct rPPG in real-world applications, it is necessary to constrain face motion or to use an rPPG algorithm that can compensate for face motion, such as the method proposed by Wang et al. [16]. The method estimates the plethysmographic signal robustly against body motion by simultaneously sampling pulse signals from multiple regions. Therefore, one future goal is to develop a method that can efficiently exclude body motions in order to minimize the effects of motion artifacts.

## 5. Conclusions

This study examined the effects of lighting environment, camera frame rate, and body motion on the accuracy of RRI and HRV measurements using rPPG in the context of telemedicine. The results show that the mean absolute error of the RRI was minimized when 500–700-lux light was shone from the front. In addition, HRV showed a high correlation coefficient when the frame rate was higher than 30 fps. These findings can set the basic requirements for not only telemedicine but also other studies using the rPPG technique. However, because the accuracy of RRI and HRV measurements are greatly affected by body motion, it is necessary to suppress body motion, especially head motion. It is also important to develop rPPG algorithms that are robust and adapt to various body motions, which would contribute to accelerating the use of rPPG in telemedicine services.

## Figures and Tables

**Table 1:** Mean absolute error (MAE) of RRI and correlation coefficient of HRV.

Condition	MAE of RRI [ms]	CORR of LF [-]	CORR of HF [-]	CORR of LF/HF [-]
FIX (fixed)	23.9	0.967	0.967	0.976
nFIX (stationary)	26.1	0.796	0.796	0.643
SPK (speaking)	53.9	0.254	0.254	0.047

## Data Availability

Not applicable.

## References

[1] Kichloo A., Albosta M., Dettloff K., Wani F., El-Amir Z., Singh J., Aljadah M., Chakinala R.C., Kanugula A.K., Solanki S. (2020). Telemedicine, the current COVID-19 pandemic and the future: A narrative review and perspectives moving forward in the USA. Fam. Med. Community Health.

[2] Ahmed Thouheed S.S., Thanuja K., Guptha N.S., Narasimha S. Telemedicine approach for remote patient monitoring system using smart phones with an economical hardware kit. Proceedings of the International Conference on Computing Technologies and Intelligent Data Engineering Conference (ICCTIDE’16).

[3] Yamazaki Y., Ishii M., Ito T., Hashimoto T. (2021). Frailty Care Robot for Elderly and its Application for Physical and Psychological Support. J. Adv. Comput. Intell. Intell. Inform..

[4] Remote Patient Monitoring for Diabetes and Related Chronic Conditions Glooko. https://www.glooko.com.

[5] Abo-Zahhad M., Ahmed M.A., Elnahas O. (2014). A Wireless Emergency Telemedicine System for Patients Monitoring and Diagnosis. Int. J. Telemed. Appl..

[6] Kamath M.V., Ghista D.N., Fallen E.L., Fitchett D., Miller D., McKelvie R. (1987). Heart rate variability power spectrogram as a potential noninvasive signature of cardiac regulatory system response, mechanisms, and disorders. Heart Vessel..

[7] Moriguchi A., Otsuka A., Kohara K., Mikami H., Katahira K., Tsunetoshi T., Higashimori K., Ohishi M., Yo Y., Ogihara T. (1992). Spectral change in heart rate variability in response to mental arithmetic before and after the beta-adrenoceptor blocker, carteolol. Clin. Auton. Res..

[8] Verkruysse W., Svaasand L.O., Nelson J.S. (2008). Remote plethysmographic imaging using ambient light. Opt. Express.

[9] Liu H., Wang Y., Wang L. A review of non-contact, low-cost physiological information measurement based on photoplethysmographic imaging. Proceedings of the 34th Annual International Conference of the IEEE Engineering in Medicine and Biology Society Conference (EMBC 2012).

[10] Takano C., Ohta Y. (2007). Heart rate measurement based on a time-lapse image. Med. Eng. Phys..

[11] Alian A.A., Shelley K.H. (2014). Photoplethysmography. Best Pract. Res. Clin. Anaesthesiol..

[12] Tarassenko L., Villarroel M., Guazzi A., Jorge J., A Clifton D., Pugh C. (2014). Non-contact video-based vital sign monitoring using ambient light and auto-regressive models. Physiol. Meas..

[13] Vogels T., van Gastel M., Wang W., de Haan G. Fully-automatic camera-based pulse-oximetry during sleep. Proceedings of the 2018 IEEE/CVF Conference on Computer Vision and Pattern Recognition Workshops (CVPRW2018).

[14] Cobos-Torres J.-C., Abderrahim M., Martinez-Orgado J. (2018). Non-contact, simple neonatal monitoring by photoplethysmography. Sensors.

[15] Papageorgiou A., de Haan G. (2014). Adaptive Gain Tuning for Robust Remote Pulse Rate Monitoring under Changing Light Conditions. Master’s Thesis.

[16] Wang W.W., Stuijk S., De Haan G.G. (2014). Exploiting Spatial Redundancy of Image Sensor for Motion Robust rPPG. IEEE Trans. Biomed. Eng..

[17] Ethan B., Justin E. Effects of frame rate and image resolution on pulse rate measured using multiple camera imaging photoplethysmography. Medical Imaging 2015: Biomedical Applications in Molecular, Structural, and Functional Imaging.

[18] Poh M.-Z., McDuff D.J., Picard R.W. (2010). Advancements in Noncontact, Multiparameter Physiological Measurements Using a Webcam. IEEE Trans. Biomed. Eng..

[19] De Haan G., Jeanne V. (2013). Robust pulse rate from chrominance-based rppg. IEEE Trans. Biomed. Eng..

[20] De Haan G., Jeanne V. (2014). Improved motion robustness of remote-PPG by using the blood volume pulse signature. Physiol. Meas..

[21] Moco A.V., Stuijk S., de Haan G. (2016). Ballistocardiographic Artifacts in PPG Imaging. IEEE Trans. Biomed. Eng..

[22] Camm A.J. (1996). Heart rate variability Standards of measurement, physiological interpretation, and clinical use. Eur. Heart J..

[23] Kumar M., Veeraraghavan A., Sabharwal A. (2015). DistancePPG: Robust non-contact vital signs monitoring using a camera. Biomed. Opt. Express.

[24] Wang W., Brinker A., Stuijk S., de Haan G. (2017). Algorithmic principles of remote PPG. IEEE Trans. Biomed. Eng..

[25] Baltrusaitis T., Zadeh A., Lim Y.C., Morency L.-P. OpenFace 2.0: Facial Behavior Analysis Toolkit. Proceedings of the 13th IEEE International Conference on Automatic Face & Gesture Recognition Conference (FG 2018).

[26] Vezhnevets V., Sazonov V., Andreeva A. A Survey on Pixel-Based Skin Color Detection Techniques. Proceedings of the International Conference on Computer Graphics and Vision Conference.

[27] Lin Y., Yuan-Hsiang L. A study of color illumination effect on the SNR of rPPG signals. Proceedings of the 39th Annual International Conference of the IEEE Engineering in Medicine and Biology Society Conference (EMBC2017).

[28] Pai A., Veeraraghavan A., Sabharwal A. (2018). CameraHRV Robust measurement of Heart Rate Variability using a Camera. Opt. Diagn. Sens. XVIII: Towar. Point-Care Diagn..

[29] Tarvainen M.P., Niskanen J.-P., Lipponen J.A., Ranta-Aho P.O., Karjalainen P.A. (2014). Kubios HRV–Heart Rate Variability Analysis Software. Comput. Methods Programs Biomed..

[30] Wang W., den Brinker A.C., Stuijk S., de Haan G. (2017). Robust heart rate from fitness videos. Physiol. Meas..

[31] Van Gastel M., Stuijk S., de Haan G. (2015). Motion robust remote-PPG in infrared. IEEE Trans. Biomed. Eng..

[32] Sun Y., Hu S., Azorin-Peris V., Kalawsky R., Greenwald S. (2013). Noncontact imaging photoplethysmography to effectively access pulse rate variability. J. Biomed. Opt..

